# Associations of IL-12, IL12R polymorphisms and serum IL-12 levels with high-risk human papillomavirus susceptibility in rural women from Luohe, Henan, China

**DOI:** 10.1097/MD.0000000000016991

**Published:** 2019-09-20

**Authors:** Jiayu Song, Qingwei Zhang, Rong Wang, Mingzhen Sun, Shaoju Jin

**Affiliations:** a Department of Pharmacology, Luohe Medical College; b Department of Obstetrics and Gynecology, Luohe Central Hospital, Luohe, Henan; c Department of Nuclear Medicine, General Hospital of Ningxia Medical University, Yinchuan, Ningxia; d Tumor Occurrence and Prevention Research Innovation Team of Henan, Luohe, Henan, China.

**Keywords:** high-risk human papillomavirus, IL-12, IL12R, polymorphisms, susceptibility

## Abstract

**Background:**

Interleukin 12 (IL-12) and interleukin 12 receptor (IL12R), key inflammatory cytokines in the immune system, participate in bridging the innate immunity and adaptive immunity. No previous work has reported the role of IL-12 and IL12R in high-risk human papillomavirus (hrHPV) susceptibility. The purpose of this study was to investigate the association of IL-12, IL12R polymorphisms, and serum IL-12 levels with hrHPV susceptibility in rural women from Luohe, Henan, China.

**Methods:**

Two hundred sixty cases with hrHPV infection and 260 healthy controls were selected. Enzyme-linked immunosorbent assays were used to detect the serum IL-12 levels, and the polymorphisms of IL12B rs3212227, IL12RB1 rs393548, and IL12RB1 rs436857 were determined using DNA sequencing.

**Results:**

The serum IL-12 levels were significantly lower in cases with hrHPV infection compared with those in healthy controls (*P* < .01).There was no significant difference in IL12 rs3212227, IL12RB1rs436857, and IL12RB1rs393548 genotype and allele frequencies between cases and controls (*P* > .05). Furthermore, with respect to the IL12 rs3212227 polymorphism with serum IL-12 levels, although serum IL-12 levels were lower in cases than in controls, we did not find any differences between serum IL-12 levels and genotypes in cases(*P* > .05).

**Conclusions:**

Our data demonstrates that low serum IL-12 levels may be associated with hrHPV susceptibility but are not associated with IL-12 gene polymorphisms; furthermore, IL-12 and IL12R gene polymorphisms may not contribute susceptibility to hrHPV in rural women from Luohe, Henan, China.

## 1 Introduction

Cervical cancer is the second most common cancer in women worldwide,^[1]^ with more than 85% of the cases occurring in developing countries.^[2,3]^ Almost 90% of cervical cancer cases are caused by human papillomavirus (HPV) infection,^[4,5]^ so HPV is considered to be an important carcinogen in women.^[6,7]^ Genital tract infection by HPV is very common, with over 80% of women infected at some point in their lives, but the majority (80–90%) can clear the virus on their own without symptoms.^[8,9]^ Few of those with persistent infection eventually progress to carcinoma.^[10]^

The host immune response is crucial to resist HPV infection and clearance.^[11]^ The failure of the immune response and imbalance secretion of inflammatory cytokines play a critical role in HPV persistence and subsequent tumorigenesis.^[11,12]^ Interleukin12 (IL-12), a key inflammatory cytokine in the immune system, participates in connecting the innate immunity and adaptive immunity.^[13,14]^ Both IL-12 and IL-12 receptor (IL12R) genes are key immune response genes. IL-12 is composed of p35 and p40 subunits, which are encoded by the IL12A and IL12B genes respectively.^[15]^ The IL12B gene on chromosome 5 may affect the production of IL12,^[16]^ which plays a critical role in the elimination of virus-infected cells.^[17]^ IL12R is composed of two subunits, which are encoded by the IL12RB1 and IL12RB2 genes.^[18]^ The IL12RB1 gene is located on chromosome 19p13.1 and encodes the ligand-binding chain of IL12R.^[19]^ Genetic variations in the IL-12 and IL12R genes were associated with viral infection, such as hepatitis B virus (HBV) and hepatitis C virus (HCV).^[17,20]^

Yang et al reported that an increased level of IL-12 was associated with high-risk human papillomavirus (hrHPV) positivity,^[21]^ but Bais et al found that there was no significantly increased in hrHPV-positive women.^[22]^ So, up to now, it is not clear whether the expression level of IL-12 is related to hrHPV susceptibility. In this study, in order to further investigate the role of IL-12 and IL-12R in the susceptibility of hrHPV in rural women from Luohe, Henan, China, we detected the expression levels of IL-12 in serum from 260 cases with hrHPV infection and 260 healthy controls. Moreover, the single-nucleotide polymorphisms (SNPs) of IL12B and IL12RB1 were tested. To the best of our knowledge, this study found a direct correlation between serum IL12 levels and hrHPV infection, and no association between the polymorphisms of IL12B rs3212227, IL12RB1 rs393548, IL12RB1 rs436857, and HPV susceptibility.

## 2 Materials and methods

### 2.1 Study population

This case-control population study was hospital-based and consisted of 260 hrHPV infection cases and 260 healthy controls. The subjects were enrolled from cervical cancer screening in rural areas of Henan province, China between September 2015 and December 2016. The inclusion criteria were:

(1) age ranging from 25 to 65 years;
(2) a sexual history of more than 3 years;
(3) 72 hours after menstruation;
(4) 72 hours after sexual activity or vaginal medication.

The exclusion criteria included having a medical history of cervical disease, uterectomy, immunological diseases, transplantation, and malignant tumor radiotherapy. The controls were matched with the cases in terms of age, nationality, initial birth age, number of pregnancies and number of births at a ratio of 1:1. The protocol was approved by the Ethics Committee of the First Affiliated Hospital of Luohe Medical College, and written informed consent was obtained from each participant before the study.

### 2.2 HPV infection testing

A cytobrush was rotated softly in the ectocervix to take specimens, and then the head of the brush was placed in small bottle containing preserving fluid stored in a refrigerator at a temperature of 4°C until detection. The DNA of 15 high-risk HPV types (16, 18, 31, 35, 39, 45, 45, 55, 52, 53, 56, 58, 59, 59, 69, 66, 68) was semi-quantitatively examined according to the manufacturer's instructions of the HPV Geno Array test kit (Zhong sheng Fang zheng Bio-technology Co., Ltd. Jiangsu, China).

### 2.3 Genotyping

Five milliliters of venous blood was collected from each participant in EDTA anticoagulant tubes, and preserved at −80°C. Genomic DNA was extracted from white blood cells using the Blood Genomic DNA Extraction kit (Takara, Japan), according to the manufacturer's instructions and was amplified by polymerase chain reaction (PCR). The information of primers and PCR conditions were described in Table 1. To confirm the genotyping results, PCR-amplified DNA samples were detected by DNA sequencing.

**Table 1:**
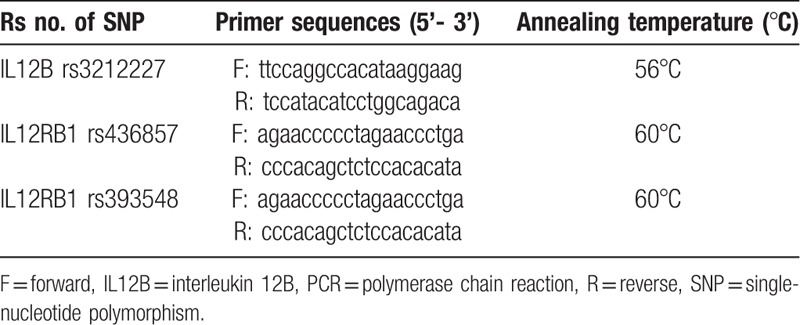
Summary of primer andPCR reaction conditions.

### 2.4 Enzyme-linked immunosorbent assay

Serum levels of IL12B were measured by enzyme-linked immunosorbent assay kits (Sunredbio, Shanghai, PRC) following the instructions of the manufacturer and expressed as nanograms per liter of serum.

### 2.5 Statistical analysis

SPSS 22.0 software (SPSS, Chicago, IL) was used for statistical analysis. The differences of demographic characteristics between the 2 groups were tested by the Student's *t* test and chi-squared test. The deviation from Hardy–Weinberg equilibrium (HWE) was also performed using the chi-squared test. The odds ratios (ORs) and 95% confidence intervals (CIs) were applied to measure the associations between genetic polymorphisms and the risk of HPV infection. The statistical significance of serum IL-12B levels in cases and controls was estimated by the Student's *t* test. Serum IL-12B levels were compared with analysis of variance between different genotypes. *P* < .05 was considered a significant difference.

## 3 Results

### 3.1 Demographic characteristics of the subjects

The demographic characteristics of the cases and controls in this study are summarized in Table 2. The cases and controls were matched since there were no significant differences in terms of age, nationality, initial birth age, number of pregnancies, and number of births between two groups (*P* > .05).

**Table 2:**
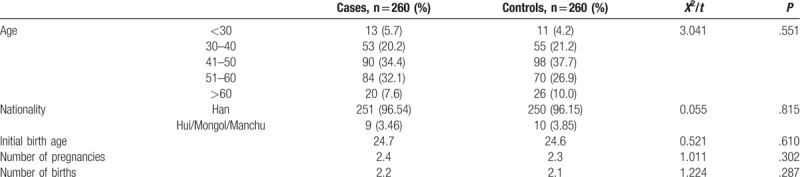
Demographic characteristics between cases and controls.

### 3.2 Genotyping results

The genotype and allele frequencies of IL12B rs3212227R, IL12RB1 rs436857, IL12RB1 rs393548 between cases and controls are shown in Table 3. The genotype distributions in the control group were all consistent with Hardy–Weinberg equilibrium (*X*^2^ = 0.126, *P* = .939; *X*^2^ = 0.255, *P* = .880; *X*^2^ = 0.553, *P* = .758). However, genotype and allele frequencies of IL12B rs3212227, IL12RB1 rs436857, and IL12RB1rs393548 were not associated with hrHPV susceptibility (*P* > .05).

**Table 3:**
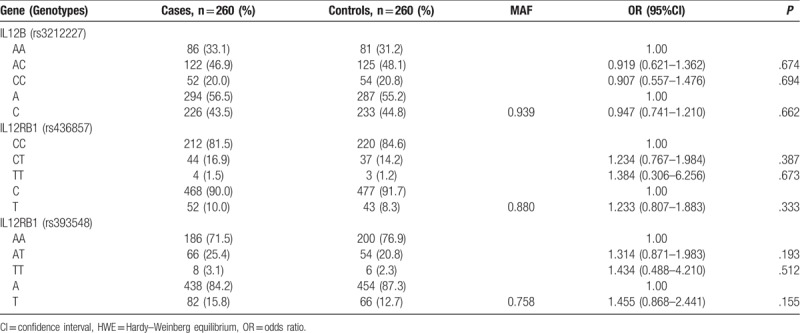
Genotype and allele frequencies between cases and controls.

### 3.3 Serum IL-12B levels

**Figure 1:**
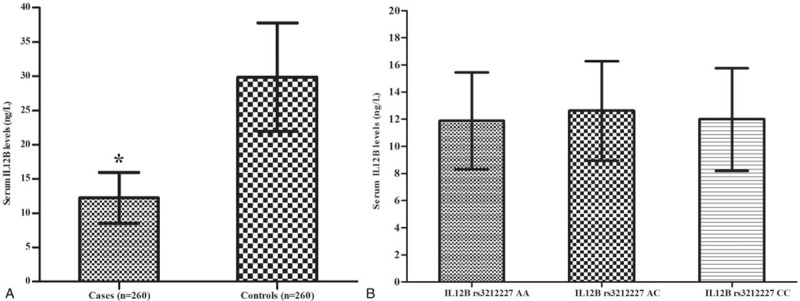
(A) Serum IL12B levels between cases and controls (∗*P* < .05). (B) Serum IL12B levels in different genotypes of IL12B rs3212227 in cases. Data were assessed using apaired *t* test and analysis of variance and presented as mean ± SD. IL12B = interleukin12B, SD = standard deviation.

As shown in Figure 1A, the serum IL-12B levels were significantly decreased incases (12.237 ± 3.738 ng/L) compared with those in controls (29.864 ± 7.926 ng/L, *P* < .01). Figure 1B shows that the expression of serum IL-12B levels with IL12B rs3212227 TT/TG/GG genotype in the case group was 11.869 ± 3.567, 12.606 ± 3.677, and 11.980 ± 3.777, respectively. No significant differences of the IL-12B gene polymorphisms were detected with respect to serum IL-12B levels in cases (Fig. 1B).

## 4 Discussion

HPV infection is very common among women. Approximately 80% to 90% of those infected with HPV spontaneously clear the virus in 12 to 24 months without showing any symptoms, while some individuals experience persistent HPV infection.^[8,9]^ These phenomena suggest that individual differences in immune responses may affect the occurrence and outcome of HPV infection.^[23,24,25]^ However, the exact mechanism associated with the occurrence and clearance of HPV infection remains uncertain. In this study, we investigated whether the IL12 rs3212227, IL12RB1rs436857, and IL12RB1rs393548 polymorphisms were associated with the susceptibility of hrHPV in rural women from Luohe, Henan, China.

Both the innate immunity and adaptive immunity are involved in the clearance, persistence, or progression of HPV infection.^[26]^ As the first line of defense to resist the invasion of HPV, the innate immunity can adjust the host reactivity and reduce the replication and transmission of HPV before the adaptive immunity is established.^[13,27]^ Adaptive immunity is composed of 2 systems: type 1 helper T cells (Th1) response and type 2 helper T cells 2 (Th2) response, and Th1 response plays a pivotal role in eliminating HPV infection.^[27–29]^

As a Th1 proinflammatory cytokine, IL-12 regulates the maturation of Th1 cells from the naive CD4+ T cell,^[30,31]^ exhibits an immunoregulatory impact on T and NK cells by inducing IFN-γ biosynthesis,^[32,33]^ and plays an important role in bridging the innate immunity and adaptive immunity.^[13,14]^ Therefore, IL-12 might promote the clearance of viral infection and reduce the chances of persistent viral infection.^[34,35]^ Studies have shown that during viral infection, the expression of cytokines was changed and the serum levels of IL-12 were decreased.^[17]^ The clearance of the virus is highly dependent on a substantial increase in the production of IL-12.^[36,20]^ Our study found that the expression of IL-12 decreased significantly in cases with hrHPV infection, indicating that low serum IL-12 levels were related to the hrHPV infection.

IL12 polymorphism located in the 3′- untranslated region (3′- UTR) at +1188A/C (rs3212227) may regulate the expression pattern of cytokines and then affect the susceptibility of viral infection. Previous studies have found that the IL12 rs3212227 CC genotype is associated with enhanced IL-12 production^[37]^ and helps the host's defense against viral infection and clearance.^[20]^ However, little is known about the association of genetic polymorphisms of IL-12 with hrHPV susceptibility. According to our results, there was no significant difference in IL12 rs3212227, IL12RB1 rs436857, and IL12RB1 rs393548 genotypes and allele frequencies between the cases with hrHPV infection and the healthy controls. Furthermore, with respect to the IL-12 rs3212227 polymorphism with serum IL-12 levels, although serum IL-12 levels were lower in cases than in controls, we also did not find any differences between serum IL-12 levels and IL-12rs3212227 polymorphisms in cases, suggesting that different genotypes might not influence the production of IL-12. Hence, we report that the polymorphisms of IL12 rs3212227, IL12RB1 rs436857, and IL12RB1 rs393548 are not associated with hrHPV susceptibility in rural women from Luohe, Henan, China.

## 5 Conclusion

In conclusion, our data demonstrates that low serum IL-12 levels may be associated with hrHPV susceptibility, but are not associated with IL-12 gene polymorphisms; furthermore, IL-12 gene polymorphism may not contribute to susceptibility to hrHPV in rural women from Luohe, Henan, China. However, we only investigated 3 SNPs for hrHPV susceptibility with limited samples. Further investigation into the relationship between SNPs and hrHPV susceptibility is needed.

## Author contributions

**Resources:** Jiayu Song, Qingwei Zhang, Rong Wang, Mingzhen Sun, Shaoju Jin.

**Formal analysis:** Jiayu Song, Mingzhen Sun, Rong Wang, Shaoju Jin.

**Writing – original draft:** Jiayu Song, Qingwei Zhang.

**Writing – review & editing:** Jiayu Song, Shaoju Jin.

**Formal analysis:** Jiayu Song, Rong Wang, Mingzhen Sun, Shaoju Jin.

**Resources:** Jiayu Song, Qingwei Zhang, Rong Wang, Mingzhen Sun, Shaoju Jin.

**Writing – original draft:** Jiayu Song, Qingwei Zhang.

**Writing – review & editing:** Jiayu Song, Shaoju Jin.
